# Effective Coverage in Health Systems: Evolution of a Concept

**DOI:** 10.3390/diseases11010035

**Published:** 2023-02-22

**Authors:** Aliya Karim, Don de Savigny

**Affiliations:** 1 Swiss Tropical and Public Health Institute, 4123 Allschwil, Switzerland; 2 University of Basel, 4001 Basel, Switzerland

**Keywords:** intervention coverage, effective coverage, community effectiveness, systems effectiveness, quality of care, cascade of care, visualization, health system performance

## Abstract

The manner in which high-impact, life-saving health interventions reach populations in need is a critical dimension of health system performance. Intervention coverage has been a standard metric for such performance. To better understand and address the decay of intervention effectiveness in real-world health systems, the more complex measure of “effective coverage” is required, which includes the health gain the health system could potentially deliver. We have carried out a narrative review to trace the origins, timeline, and evolution of the concept of effective coverage metrics to illuminate potential improvements in coherence, terminology, application, and visualizations, based on which a combination of approaches appears to have the most influence on policy and practice. We found that the World Health Organization first proposed the concept over 45 years ago. It became increasingly popular with the further development of theoretical underpinnings, and after the introduction of quantification and visualization tools. The approach has been applied in low- and middle-income countries, mainly for HIV/AIDS, TB, malaria, child health interventions, and more recently for non-communicable diseases, particularly diabetes and hypertension. Nevertheless, despite decades of application of effective coverage concepts, there is considerable variability in the terminology used and the choices of effectiveness decay steps included in the measures. Results frequently illustrate a profound loss of service effectiveness due to health system factors. However, policy and practice rarely address these factors, and instead favour narrowly targeted technical interventions.

## 1. Introduction

The manner in which high-impact, life-saving health interventions reach populations in need is a critical dimension of health system performance. Indeed, the measurement of intervention “coverage” has been one of the longest-established health metrics in public health, especially in low- and middle-income countries. A classic example of a coverage metric is the cluster sample survey of immunization coverage for highly cost-effective vaccines used by the expanded immunization program (EPI) since the late 1970s [1]. Surveys of intervention access and coverage remain today as core components of significant omnibus surveys such as the national Demographic and Health Surveys (DHS), the UNICEF Multiple Indicator Cluster Surveys (MICS), the WHO Service Availability and Readiness Assessment (SARA) surveys, and the ICF Service Provision Assessment (SPA) surveys. These are conducted in line with guidelines and standards developed by WHO technical programs and the work of the International Health Facility Assessment Network (IHFAN).

Global health has long recognized that such supply side surveys of access, availability, and affordability as coverage drivers are insufficient, and we need to more fully unpack the demand side. The demand side includes perceptions of the health system, the human behavioural aspects of health providers in adequately providing the intervention compliant with standards, and the human behaviour of clients of the health system based on the acceptability of and adherence to the intervention. This helps explain the long-running frustration of development partners that the efficacy of health interventions, as proven in Phase IV research trials in ideal circumstances, often fails to translate to effectiveness at the population level, even when coverage is high. Effectiveness is defined as “a measure of the extent to which a specific intervention, procedure, regimen, or service, when deployed in the field in routine circumstances, does what it is intended to do for a specified population” [2].

To better understand and address the decay of efficacy to effectiveness in real-world health systems, the more complex measurement of “effective coverage”, which includes coverage and the potential health gain the health system could deliver, is required. That is to say, the proportion of a population needing an intervention or service that benefits from a positive outcome [3,4]. Early development and continued promotion of the basic concept of effective coverage in low- and middle-income countries were heavily driven and influenced by the efforts of Marcel Tanner and colleagues at the Swiss Tropical and Public Health Institute [5,6,7,8,9,10,11,12,13,14,15].

Measuring effective coverage is complex and costly. Approaches vary with the intervention or service addressed and the effect it targets. The indicators used in the older Millennium Development Goals (MDGs) and Universal Health Coverage (UHC) have had a simple coverage focus. This has shifted under the newer Sustainable Development Goals toward effective coverage [16]. The SDG Countdown to 2030 initiative has a Coverage Technical Working Group promoting improved metrics focused on effective coverage [17].

Despite over 20 years of publications on various dimensions of effective coverage, we still need to understand how well such evidence has influenced health policies and systems’ improvement. A recent systematic review has found profound heterogeneity in the terminology, definitions, and conceptualization of effective coverage. Despite the current popularity of coverage efficacy to effectiveness cascades, there is considerable variability and nuance in the terminology used and the choices of cascade steps included [18].

The rationale for this narrative review is to trace back the origins, timeline, and evolution of the conceptual thinking behind effective coverage metrics to illuminate potential improvements in coherence, terminology, application, and visualizations, based on which approaches appear to have the most influence on policy and practice.

## 2. Materials and Methods

### 2.1. Review Approach

We have chosen a narrative (literature) review approach to provide a comprehensive overview of the evolution of effective coverage to contribute to a theoretical framing and context for a way forward. We subscribe to the standards of narrative reviews proposed by Green et al. (2006) [19]. We complied with the norms prescribed in their methodological approach, including standards pertaining to the sources of information, search terms, selection criteria and delimiting parameters, and conceptual synthesis. As a quality control measure, we corroborated our methods with the SANRA scale [20] to guide the review process.

We searched PubMed with the last update on 6 December 2022, without publication date restrictions. We developed a code bank of search terms deemed relevant to this review (Table 1). We identified these based on their association with systems’ effectiveness, effective coverage, and the cascade or trajectory of care. Additionally, we subsequently searched publications citing identified milestone papers.

### 2.2. Content Analysis

We used Zotero 6.0 software (Corporation for Digital Scholarship, Vienna, VA, USA) to import all results retrieved from the search. Following duplicate removal, publications were screened according to generalized inclusion and exclusion criteria. They were excluded if studies were not published in English or German, or if they focused on areas outside of health sciences. Studies describing Markov chain models were excluded due to their stochastic nature and lack of sequential dependency on multiple previous states. While we aim to track the path of this concept over time and its crossover into the public health literature from other domains, we focus primarily on public health and its associated interventions in low- and middle-income countries. This resulted in a final selection of 244 publications based on their methods and their historical and conceptual relevance. The exclusion of other relevant publications in this review thus does not preclude their association with or treatment of the concepts of effective coverage, systems effectiveness, or the cascade of care. For discussion, we further selected a small number of “milestone papers” as those that we considered were the first papers in the timeline that advanced the concept by adding value through additional conceptualization, terminology, statistical approach, method of visualization, or novel application.

## 3. Results

### 3.1. Literature Retrieved

We provide all search terms, the number of publications retrieved, and the number relevant to the effective coverage concept, especially as applied in low- and middle-income countries, in Table 1.

We discuss the selected results profiled as milestone papers in Section 3.2, and provide exemplars of novel visualizations in Appendix A.

### 3.2. Main Milestones in the Timeline of the Effective Coverage Concept

Where and when did the concept of effective coverage originate, and how has it evolved over the years? Our review traces the origins of the basic idea of effective coverage to a seminal paper in 1978 by Dr. T. Tanahashi at the WHO [21]. Tanahashi noticed a problem with the usual approach to coverage that focused narrowly on geographic and population access as a principle to justify resource allocation for policymakers. As an alternative, he proposed to address the concept of “health service coverage” by adding five key measures [22], reflecting different stages along the service provision continuum. The elements he suggested were availability of service, quality of coverage, accessibility, initial contact with the health system, and continued utilization. 

Tanahashi’s paper languished in citation obscurity for 30 years. Without metrics, the concept had little traction in the literature until 2012 [22], when it suddenly started to accumulate dozens of citations. During its obscurity, in 1985, Peter Tugwell and colleagues in Canada working on health technology assessment independently proposed the concept of “community effectiveness” [23], without reference to Tanahashi but with a similar conceptualization. The Tugwell team introduced the added dimension of measurement to effective coverage. This was also the first paper to express measures of intervention performance in health systems in terms of sequential conditional probabilities of health system factors that could erode the efficacy of an intervention. They proposed integrating five measures: coverage, screening or diagnostic accuracy, health provider compliance with intervention guidelines, patient compliance or adherence, and efficacy. Each of these elements constitutes hurdles across the trajectory of care that beneficiaries must clear in sequence to benefit from the intervention’s efficacy. Subsequently, the term “effective coverage” started to appear in the literature to reflect this same notion, but did not yet include all the dimensions introduced by Tugwell [24,25].

The next milestone for understanding, measuring, and documenting effective coverage was the graphical visualization of the cascading effect of the Tanahashi and Tugwell elements produced in 1990 by Marcel Tanner [5] and Vlassoff and Tanner [6]. They introduced a staircase graphic of Tugwell’s community effectiveness, illustrating the incremental effects of coverage, diagnostic accuracy, provider compliance, user adherence, and efficacy on community effectiveness. They were also the first to point out that intervening piecemeal on only one or two elements has little effect on final community effectiveness.

The development of the WHO World Health Report 2000 [26] prompted a wealth of debate and the development of approaches to measurement of health systems’ performance. This, in turn, led to deliberations at the conceptual level on “effective coverage”, both in the WHO and globally. Chris Murray and David Evans conducted and summarized these debates from 2000 to 2003 [3]. Shengelia et al. at the WHO in 2005 [27] later integrated this into a conceptual framework and measurement strategy. Shengelia was the first to use the term ‘effective coverage’ in the title of a paper, but does not cite any earlier literature reported here.

Rafael Lozano led the first significant use of these new metrics for multiple interventions to determine the effective coverage of 18 common health services in order to benchmark comparative sub-national (state-level) health system performance in Mexico [4]. The definition of effective coverage used in this first practical application was the “proportion of potential health gain that the health system could deliver to that which is delivered”. This led to the rapidly increasing application of effective coverage studies in Mexico and elsewhere.

The effective coverage concept found additional mathematical footing within conditional probability theory and survival analysis methods evolving into tree-structured survival models [28,29]. The latter uses a class of nonparametric regression modelling techniques that enable a more flexible assessment of covariates across a series of sequential binary outcomes. Variants of this approach have previously been applied in clinical contexts and health economics research, but less frequently for targeted health interventions. In 2003, Lemon et al. synthesized a proposed public health application of recursive partitioning or classification and regression tree analysis as a meaningful approach to assessing population outcomes, according to group profiles across a trajectory of care [30]. 

In 2006, Tugwell et al. [9] augmented their community effectiveness concept by introducing an equity dimension termed “equity effectiveness”. They examined the differentials occurring when effective coverage results are disaggregated by socioeconomic status, showing that the decay of high efficacy to low effectiveness is much more pronounced in services for the poorest than for the least poor.

From 2006 onward, the general concept of effective coverage started to be expressed in the literature under increasingly varied terminologies, without necessarily referring to effective coverage. Many kept the notion of a staircase or cascade depiction to assess the quality of care. For example, there was the “critical care cascade” in a systems approach by Gosh [31], and the “cascade of care” approach by Watson-Jones et al. [32] to describe attrition in the care of HIV-positive pregnant women in Tanzania. The “cascade of care” is the term now most frequently used for the concept of effective coverage for HIV, TB, Hepatitis C, Cancer and NCDs, and interventions related to substance abuse.

Recognizing the importance of health system factors driving effective coverage, several authors started to use the term “systems effectiveness” [11,12,33,34] for malaria interventions. The latest global Malaria Eradication Research Agenda (malERA) initiative in 2017 realized that they could not achieve eradication without harnessing the health system. It introduced the term “effectiveness decay” with an associated staircase graphic [35] to draw attention to the five elements of effective coverage and their impacts on the performance of highly efficacious technical interventions. 

Regarding major global goals, Murray et al. in 2019 applied the concept to model the effective coverage of Universal Health Coverage in 204 countries [36]. The global Countdown to 2030 initiative, in support of measuring progress toward the Sustainable Development Goals, has also adopted effective coverage as a core metric. It has continued to push methodological development and rigor for coverage methods [17,37].

March expanded the idea of cascades with the “health service coverage cascade” in 2020 [38] with seven elements: target population; service-contact coverage; input-adjusted coverage; intervention coverage; (process) quality-adjusted coverage; user adherence-adjusted coverage; and outcome-adjusted coverage into the cascade.

**Figure 1:**
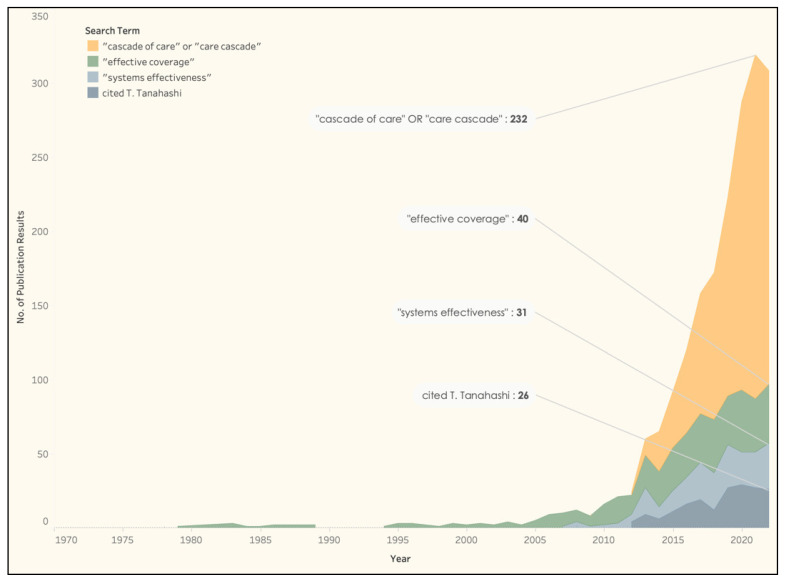
Frequency of publications relevant to effective coverage by 5-year periods since 1975. Citations of T. Tanahashi are publications referencing the landmark paper “Health Service Coverage and its Evaluation” Bull World Health Organisation 1978, 56, 295–303. Publications are indexed by PubMed as of 13 December 2022. Some results may overlap.

Recently, in 2021, with the growing panoply of terms, concepts, and measures, Exley and colleagues conducted rapid systematic reviews of effective coverage and coverage cascades in the literature since 2010 for childbirth, newborn, and child health interventions in low- and middle-income countries [18,39]. They concluded that the definitions of effective coverage differed across 64 reports on effective coverage, and varied with the number of cascade steps used to “adjust” for quality of care and the methods used to generate a composite measure. Currently, the most common conceptualization of effective coverage in the literature uses the term “cascade of care” (Figure 1). 

Various visualizations have been introduced to characterize the effective coverage concept. These have evolved from the early line graphs and histograms illustrating effectiveness decay to Vlassoff and Tanner’s staircase model in 1992 [6], and other incarnations of inverted pyramids, survival trees, and Sankey diagrams (Appendix A) [40,41,42,43,44,45,46,47,48,49,50,51]. These help to provide a compelling visual narrative to demonstrate that intervention pathways can be at once longitudinal or conditional on preceding steps, and dynamic, wherein results can change based on evolving inputs from the health system. Further, they illustrate the hazard of reliance on isolated indicators as benchmarks of performance, and draw attention to the importance of the denominators we use to measure them.

One common result of the application of the effective coverage concept is that the published results typically indicate a severe decay of effectiveness across the intervention arc. Typical results often indicate less than 20% effective coverage, despite intervention efficacies above 80% (Appendix A). What the literature rarely offers are suggestions for improving effective coverage, or studies showing deliberate health system remediation efforts aimed at improving effective coverage. This is somewhat analogous to surveillance without response.

## 4. Discussion

Effective coverage as a concept is somewhat intuitive. It is due to this simplicity that explorations of effectiveness have manifested as variations on a theme. It is so simple, in fact, that some researchers have expressed dismay at its neglect [52]. Effective coverage sits at the intersection of assessing health system outputs, implementation outcomes, the behaviour of people and institutions, the significant effects of context and policy, and the interplay of various systems’ factors across a tangible pathway of events. In so doing, it advantageously blends various conceptual approaches increasingly used in assessing interventions.

Part of our current challenge seems to stem from how we conceptualize successful implementation, and how we compartmentalize approaches to evaluation of public health programs. The former commonly leans on deliverable- or indicator-based approaches to assessment rather than answering holistic questions about sustainability, system-wide impact, and how program results fit into an overarching public health goal. Sidestepping these siloed instincts obviates the essential question: do our actions generate the impact intended?

The most common observation in the literature reviewed is how much performance loss occurs across the care trajectory of a health intervention in real-world health systems. This decay of performance is quantitatively a consequence of the conditional probabilities of even slight decays at each stage in the care cascade multiplying to result in a surprisingly large overall decay in effective coverage. The significance of these system-wide deficiencies and the importance attributable to them seem to have been ignored by health systems stewards and their funding partners in favour of a focus on improving last-mile, curative, and treatment-oriented technical approaches, rather than system-wide efforts to improve delivery and the settings that provoke poor states of health.

*Limitations of the review.* In a narrative review, there are inherent limitations. First, the selection of papers is guided by the choice of the authors, and as part of its narrative nature is not structured according to the processes of a systematic review. This can lead to the exclusion of important works that may provide additional or alternative information to what is presented here. We have taken care to mitigate this possibility. Secondly, we did not discuss all 244 papers that supported the development of this review, rather focusing on the milestone papers which characterized the evolution of the concept of effective coverage. Finally, because the concept treated in this review is based on both an intuitive and mathematical construct and the search was based on specific terminology to capture these notions, it is possible that through terminology, certain papers were excluded that may have enriched the conclusions reached.

Our review reveals the 45-year legacy of an obscure WHO publication of a concept that has since been re-invented and reincarnated many times, over decades, under many aliases and visualizations. The repeated birth of this concept in this evolution suggests that the underpinning notion, i.e., that a health intervention’s overall performance is the product (and not the sum) of many small and neglected inefficiencies in health systems, is very real. The recent exponential rise in publications, the emergence of a novel terminology around the idea of a “cascade of care”, and the new emphasis on compelling visualizations of results indicate that we may be entering an era that will focus more attention on this neglected health system problem. We need to move into an era in which new metrics, analytics, and systems’ thinking can be applied to improve the performance of intervention delivery in the health system. Additional efforts from the field of implementation science and practice will be needed. Such efforts can be directed at strengthening routine health information systems, enabling them to provide the necessary indicators to feed simple dashboards and display effective coverage results closer to real-time, and developing compelling graphics that inspire system-level strengthening. Systems strengthening can then begin to focus more on improving the health system’s ability to deliver, rather than just continuing to improve its interventions. Finally, we suggest going beyond the concept and metric of “effective coverage”, and that the term “systems effectiveness” better encompasses what we need to measure.

## Figures and Tables

**Table 1:** Search strategy and results.

Search Terms	No. of Retrieved Publications	No. of Relevant Publications
“effective coverage”	390	55
“continuum of care”	3148	25
“decision tree analysis”	912	15
“care cascade”	621	14
“bottleneck analysis”	75	13
“cascade of care”	450	13
“community effectiveness”	90	13
“tanahashi”	1431	13
“sankey diagram”	57	12
“patient dropout”	217	12
“content coverage”	260	12
“care trajectory”	299	12
“patient attrition”	153	11
“systems effectiveness”	185	9
“equity effectiveness”	27	7
“trajectory of care”	74	7
“decision tree model”	1540	5
“intervention effectiveness”	1972	5
“conditional probability”	2280	5
“quality(-)adjusted coverage”	8	4
“coverage cascade”	4	3
“effectiveness decay”	2	2
“input(-)adjusted coverage”	2	1
“efficacy decay”	5	1
“staircase model”	19	1
“effectiveness gap”	82	1
“quality-adjusted effectiveness”	4	0
“efficacy gap”	11	0
“systems coverage”	12	0
“stepwise cascade”	15	0
other identified publications		33

No results were retrieved from the following terms: “care staircase”; “care stairway”; “coverage staircase”; “dropout of cases” OR “case dropout”; “dropout of patients”; “outcome adjusted coverage”; “process(-)adjusted coverage”; “quality(-)adjusted contact”; “structure adjusted coverage”; “user(-)adjusted coverage”.

## Data Availability

No new data were created or analyzed in this study. Data sharing is not applicable to this article.

## Appendix A

Table of selected milestone papers with examples of visualizations.

**Table A1:** Select Milestone Papers and Examples of the Evolution of Effective Coverage Concepts.

Title	Author	Intervention or Disease	Decay Result *	Illustration	Citation
Health service coverage and its evaluation	Tanahashi, T	*Conceptual*	N/A		Tanahashi T. Health service coverage and its evaluation. *Bull World Health Organ*. 1978;56(2):295-303.
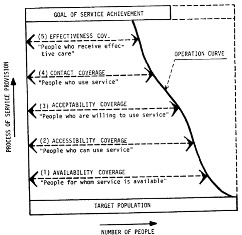
The measurement iterative loop: a framework for the critical appraisal of need, benefits and costs of health interventions	Tugwell, P, Bennett, KJ, Sackett, DL, and Haynes, RB	*Conceptual*	N/A		Tugwell P, Bennett KJ, Sackett DL, Haynes RB. The measurement iterative loop: a framework for the critical appraisal of need, benefits and costs of health interventions. J Chronic Dis. 1985;38(4):339–351. doi:10.1016/0021-9681(85)90080-3
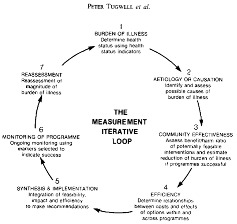
The relevance of rapid assessment to health research and interventions	Vlassoff, C, and Tanner, M	*Conceptual*	N/A		Vlassoff C, Tanner M. The relevance of rapid assessment to health research and interventions. *Health Policy Plan*. 1992;7(1):1–9. doi:10.1093/heapol/7.1.1
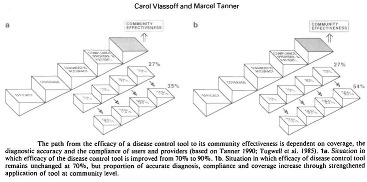
Systems effectiveness of an intervention	De Savigny, D, and Binka, F	Malaria	14%		De Savigny D. and Binka, F. INDEPTH Network INESS Project—unpublished
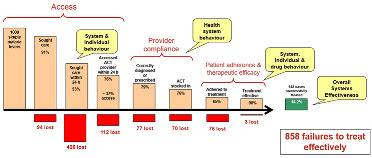
Obesity and 33-year follow-up for coronary heart disease and cancer mortality	Carmelli, D, Zhang, H, and Swan, GE	Cardiovascular disease and cancer			Carmelli D, Zhang H, Swan GE.Obesity and 33-year follow-up for coronary heart disease and cancer mortality. *Epidemiology*. 1997;8(4):378–383. doi:10.1097/00001648-199707000-00005
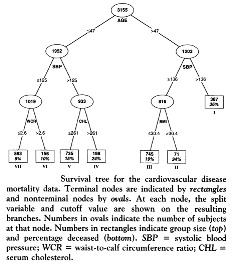
Classification and regression tree analysis in public health: Methodological review and comparison with logistic regression	Lemon, SC, Roy, J, Clark, MA, Friedmann, PD, and Rakowski, W	*Conceptual*	N/A		Lemon SC, Roy J, Clark MA, Friedmann PD, Rakowski W. Classification and regression tree analysis in public health: methodological review and comparison with logistic regression. *Ann Behav Med*. 2003;26(3):172–181. doi:10.1207/S15324796ABM2603_02
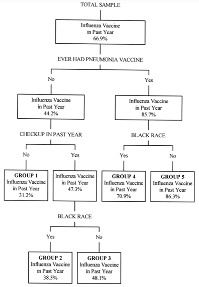
Prevalence and unmet need for diabetes care across the care continuum in a national sample of South African adults: Evidence from the SANHANES-1, 2011–2012	Stokes, A, Berry, KM, and Mchiza, Z et al.	Diabetes	19%		Stokes A, Berry KM, Mchiza Z et al. Prevalence and unmet need for diabetes care across the care continuum in a national sample of South African adults: Evidence from the SANHANES-1, 2011–2012. *PLoS One*. 2017;12(10):e0184264. Published 2017 Oct 2. doi:10.1371/journal.pone.0184264
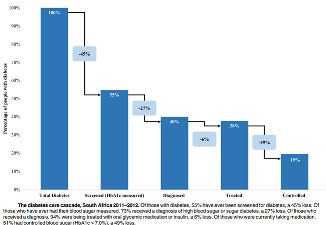
Effective Coverage and Systems Effectiveness for Malaria Case Management in Sub-Saharan African Countries	Galactionova, K, Tediosi, F, de Savigny, D, Smith, T, and Tanner, M	Malaria	8% to 72%		Galactionova K, Tediosi F, de Savigny D, Smith T, Tanner M (2015) Effective Coverage and Systems Effectiveness for Malaria Case Management in Sub-Saharan African Countries. PLoS ONE 10(5): e0127818. https://doi.org/10.1371/journal.pone.0127818
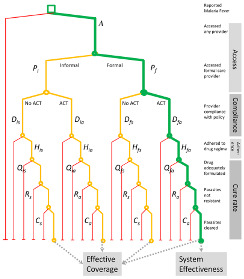
The cascade of care in diagnosis and treatment of latent tuberculosis infection: a systematic review and meta-analysis	Alsdurf, H, Hill, PC, Matteelli, A, Getahun, H, and Menzies, D	Tuberculosis	18.8%		Alsdurf H, Hill PC, Matteelli A, Getahun H, Menzies D. The cascade of care in diagnosis and treatment of latent tuberculosis infection: a systematic review and meta-analysis. Lancet Infect Dis. 2016;16(11):1269–1278. Doi:10.1016/S1473-3099(16)30216-X
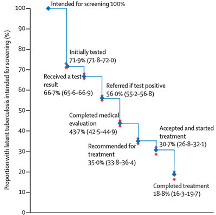
Hypertension Cascade: Hypertension Prevalence, Treatment and Control Estimates Among US Adults Aged 18 Years and Older		Hypertension			*Centers for Disease Control and Prevention (CDC). Hypertension Cascade: Hypertension Prevalence, Treatment and Control Estimates Among US Adults Aged 18 Years and Older Applying the Criteria From the American College of Cardiology and American Heart Association’s 2017 Hypertension Guideline—NHANES 2015–2018. Atlanta, GA: US Department of Health and Human Services; 2021.*
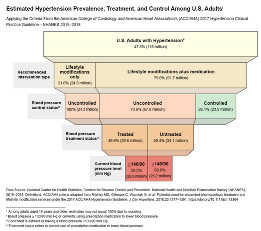
The Forest for the Trees: Effective Coverage and the Cascade of Care of Community Childhood Illness Interventions in Sub-Saharan Africa	Karim, A, et al.	Integrated community case management	0.0% to 84.6%		Authors, unpublished.
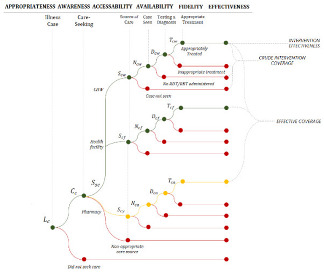
Bottlenecks in the provision of antenatal care: rural settled and mobile pastoralist communities in Chad	Lechthaler, F, Abakar, MF, Schelling, E, et al.	Antenatal care			Lechthaler F, Abakar MF, Schelling E et al.Bottlenecks in the provision of antenatal care: rural settled and mobile pastoralist communities in Chad. *Trop Med Int Health*. 2018;23(9):1033–1044. doi:10.1111/tmi.13120
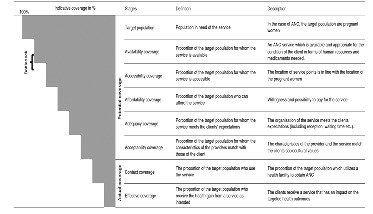
Barriers to and Facilitators of Iron and Folic Acid Supplementation within a School-Based Integrated Nutrition and Health Promotion Program among Ghanaian Adolescent Girls	Gosdin, L, Sharma, AJ, and Tripp, K et al.	Nutrition, IFA supplementation	50.4%		Gosdin L, Sharma AJ, Tripp K et al. Barriers to and Facilitators of Iron and Folic Acid Supplementation within a School-Based Integrated Nutrition and Health Promotion Program among Ghanaian Adolescent Girls. Curr Dev Nutr. 2020;4(9):nzaa135. doi:10.1093/cdn/nzaa135
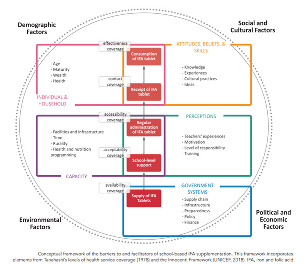
Starting at the community: Treatment seeking pathways of children with suspected severe malaria in Uganda	Brunner, N, Karim, A, and Hetzel, M et al.	Rectal artesunate (Malaria)	77.7%		Brunner N, Karim A, Hetzel M et al. Starting at the community: Treatment seeking pathways of children with suspected severe malaria in Uganda. medRxiv 2021.12.09.21267055; doi: https://doi.org/10.1101/2021.12.09.21267055
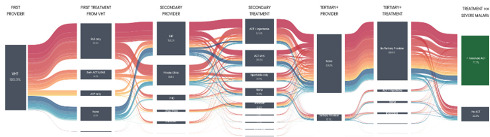
The HIV care cascade in sub-Saharan Africa: systematic review of published criteria and definitions.	Mugglin, C, Klaeger, D, and Gueler, A et al.	HIV	N/A		Mugglin C, Klaeger D, Gueler A et al. The HIV care cascade in sub-Saharan Africa: systematic review of published criteria and definitions. *Journal of the International AIDS Society*. 2021;24. doi:10.1002/jia2.25761
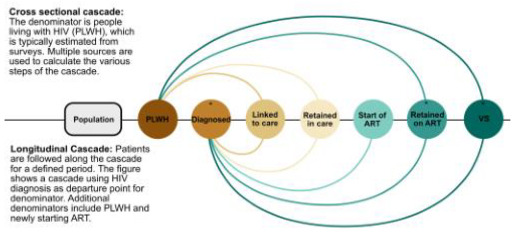
The cascade of hypertension care in Cambodia: evidence from a cross-sectional population-based survey	Chham, S et al.	Hypertension	5.7%		Chham S, Buffel V, Van Olmen J, Chhim S, Ir P, Wouters E. The cascade of hypertension care in Cambodia: evidence from a cross-sectional population-based survey. *BMC Health Serv Res*. 2022;22(1):838. Published 2022 Jun 29. Doi:10.1186/s12913-022-08232-7
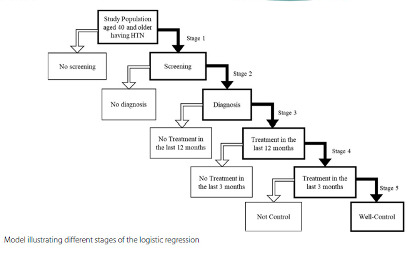
Improving services for chronic non-communicable diseases in Samoa: an implementation research study using the care cascade framework	Fraser-Hurt, N, Naseri, LT, and Thomsen, R et al.	Hypertension	4.7%		Fraser-Hurt N, Naseri LT, Thomsen R et al. Improving services for chronic non-communicable diseases in Samoa: an implementation research study using the care cascade framework. Aust N Z J Public Health. 2022;46(1):36–45. doi:10.1111/1753-6405.13113
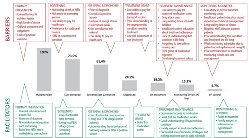

* Decay result represents the final node indicator reported in the study as a proportion of the total cases; that is, the denominator is derived from that of the first indicator reported at the beginning of the cascade. For those results representing a range, this is due to different definitions of effectiveness presented in the study, or those differentiated by demographic group, disease profile, data source or other classification.
